# Using Rule-Based Machine Learning for Candidate Disease Gene Prioritization and Sample Classification of Cancer Gene Expression Data

**DOI:** 10.1371/journal.pone.0039932

**Published:** 2012-07-11

**Authors:** Enrico Glaab, Jaume Bacardit, Jonathan M. Garibaldi, Natalio Krasnogor

**Affiliations:** 1 Interdisciplinary Computing and Complex Systems (ICOS) Research Group, University of Nottingham, Nottingham, United Kingdom; 2 Intelligent Modeling and Analysis (IMA) Research Group, University of Nottingham, Nottingham, United Kingdom; The Centre for Research and Technology, Hellas, Greece

## Abstract

Microarray data analysis has been shown to provide an effective tool for studying cancer and genetic diseases. Although classical machine learning techniques have successfully been applied to find informative genes and to predict class labels for new samples, common restrictions of microarray analysis such as small sample sizes, a large attribute space and high noise levels still limit its scientific and clinical applications. Increasing the interpretability of prediction models while retaining a high accuracy would help to exploit the information content in microarray data more effectively. For this purpose, we evaluate our rule-based evolutionary machine learning systems, BioHEL and GAssist, on three public microarray cancer datasets, obtaining simple rule-based models for sample classification. A comparison with other benchmark microarray sample classifiers based on three diverse feature selection algorithms suggests that these evolutionary learning techniques can compete with state-of-the-art methods like support vector machines. The obtained models reach accuracies above 90% in two-level external cross-validation, with the added value of facilitating interpretation by using only combinations of simple if-then-else rules. As a further benefit, a literature mining analysis reveals that prioritizations of informative genes extracted from BioHEL’s classification rule sets can outperform gene rankings obtained from a conventional ensemble feature selection in terms of the pointwise mutual information between relevant disease terms and the standardized names of top-ranked genes.

## Introduction

Gene expression profiling and data analysis is a widely used approach to gain new insights on the regulation of cellular processes in biological systems of interest. For this purpose, common statistical methods and machine learning techniques can be employed, including clustering methods to discover classes of related biological samples, feature selection methods to identify informative genes and classification methods to assign class labels to cell samples with unknown biological conditions.

Here we focus on supervised gene expression analysis of cancer microarray data using feature selection and classification methods. Further progress in the accuracy and interpretability of microarray classification models is of great practical interest, since a more accurate cancer diagnosis using microarrays would help to prevent inappropriate therapy selection.

Although high prediction accuracies have already been reached on many microarray cancer datasets, the models are often very complex and difficult to interpret, and lack robustness when being applied on external data from other experimental platforms. Specifically, challenges arise from small sample sizes, large numbers of uninformative genes, high noise levels, several outliers and systematic bias. While experiments can often be conducted with high reproducibility within a single laboratory, results obtained based on different chip technologies and experimental procedures from different laboratories are often hardly comparable. Some of these issues can be addressed by using cross-study normalization methods and integrative microarray analysis [1], [2] or by combining microarray data with clinical data [3], [4]. To obtain further improvements, in previous studies we have employed ensemble learning techniques [5]–[7] and integrated data from cellular pathways, co-expression networks and molecular interactions into the analysis [8]–[11]. However, there remains a need for more accurate, robust and easily interpretable prediction methods.

In order to alleviate some of the typical problems of current microarray studies and show the benefits of rule-based evolutionary machine learning systems for microarray sample classification, resulting from the capabilities of evolutionary computation and the enhanced interpretability of decision rules, we evaluate our previously developed machine learning systems BioHEL [12]–[15] and GAssist [16]–[20] on three large-scale, public microarray cancer datasets.

Evolutionary learning methods have already been applied successfully in different microarray studies, e.g. for selecting informative subsets of genes [21]–[23], for clustering and biclustering [24]–[26] and sample classification [27]–[29]. Moreover, in recent years new rule-based classification approaches were successfully tested on high-dimensional gene array data [30]–[33], providing human-interpretable rule sets as models.

**Figure 1:**
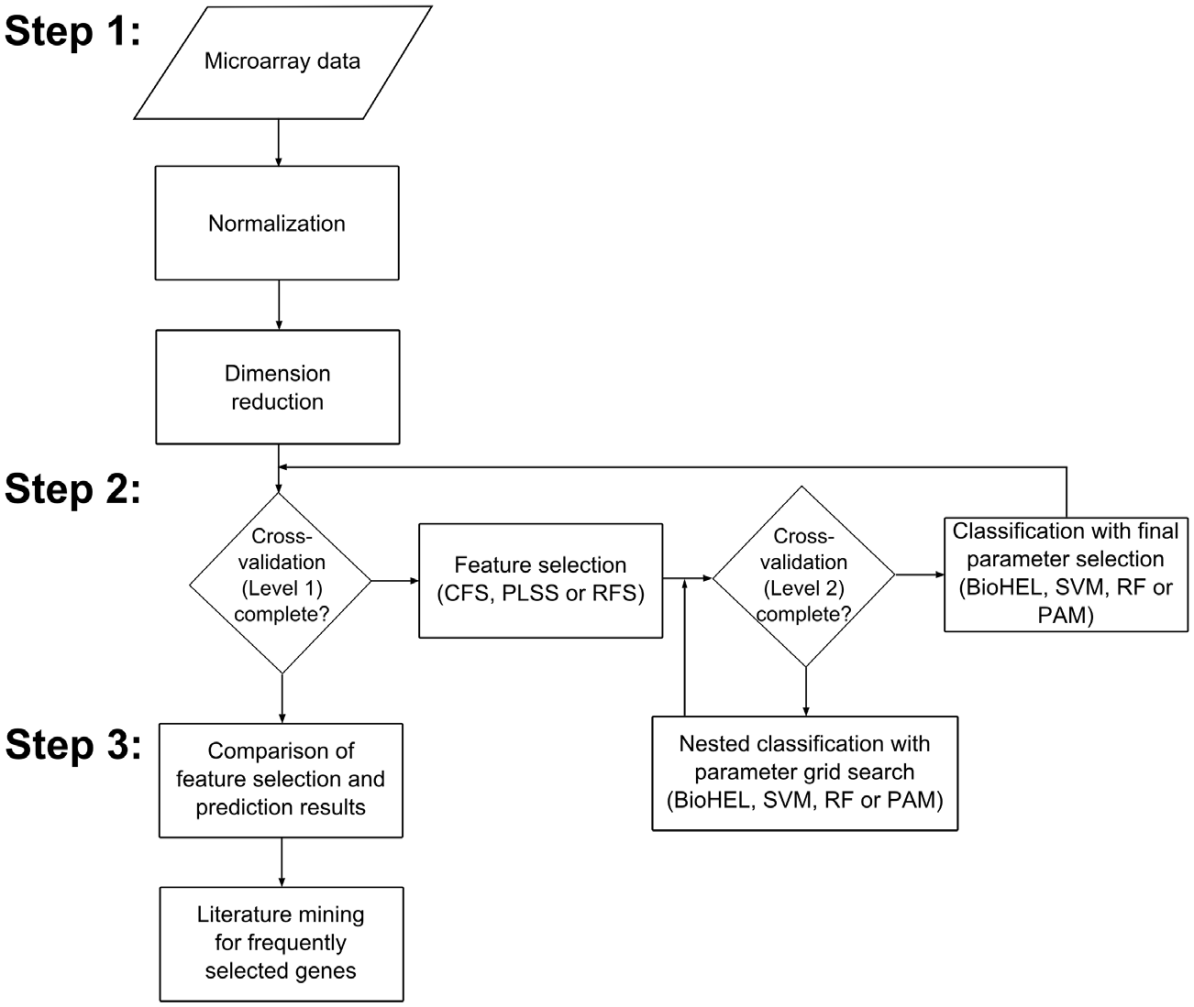
Flowchart illustrating the experimental procedure. The protocol consists of three steps: 1) Pre-processing; 2) Supervised analysis; 3) Post-analysis.

The machine learning systems presented in this paper combine these two paradigms, evolutionary search and rule learning, providing both an effective search space exploration and an enhanced model interpretability. In particular, BioHEL’s conjunctive rules can point the experimenter to potential functional association between genes [34], and its value range rules provide the user with an indication on whether a gene tends to be up- or down-regulated in the corresponding biological condition, given the complete value range across all samples. An illustration of the entire analytical protocol is shown in Fig. 1. First, we normalize each microarray dataset and pre-filter the attributes to reduce the dimensionality. Next, we apply our learning algorithms *BioHEL* [12]–[15] and *GAssist* [16]–[20] in combination with different feature selection algorithms using a cross-validation scheme and repeat this process with three alternative classifiers (see Experimental protocol). In the last step, the generated prediction results and the genetic probes (later referred to by their corresponding genes) that were considered as most informative by the learning system are analyzed statistically and using a text-mining approach to find associations between relevant disease terms and corresponding standardized gene identifiers.

We will discuss these steps in detail according to the following structure: In the Methods section we provide a step-by-step description of our experiments and explain each of the used techniques in detail, dealing first with the feature selection approaches, then with the machine learning systems BioHEL and GAssist, and finally with the datasets and pre-processing methods. The Results section contains the prediction results of running BioHEL, GAssist and the alternative classifiers on the three microarray cancer datasets. Moreover, this section presents a post-analysis of the results using biomedical literature mining. In the Conclusions section, we provide an outlook on further possible extensions of the classification framework.

In summary, the overall aim of the study was to obtain more biologically interpretable models for microarray cancer sample classification, which enable a robust prioritization of putative biomarkers and reach competitive prediction accuracies. Instead of tweaking algorithms or re-developing them from scratch to maximize accuracy at the cost of higher complexity, the goal was achieved by a new analysis pipeline that investigates how different algorithms profit from external feature selection, and that exploits the known benefits of existing evolutionary algorithms in terms of search space exploration and exploitation, and of rule-based learning methods in terms of interpretability.

## Methods

### Experimental Protocol

Our analysis pipeline to compare both feature selection and prediction methods for microarray sample classification consists of three basic steps: Data pre-processing, supervised analysis of the data and post-analysis of the results.

In the first stage, the microarray datasets are pre-processed and normalized (see section Datasets). Next, an external cross-validation is performed [35], i.e. in each cycle of the cross-validation, first a feature selection method is applied on the current training data and the resulting subset of features is used to classify the test set samples with a machine learning method. This procedure is employed using both 10-fold cross-validation (CV, with random splits but consistent splits across all comparisons) and leave-one-out CV (LOOCV) and different combinations of feature selection and classification algorithms. Specifically, the feature selection methods include the univariate filter “Partial-Least-Squares based Feature Selection” (PLSS), the combinatorial filter “Correlation-based Feature Selection” (CFS) [36] and the embedded feature selection method “Random Forest based Feature Selection” (RFS, all selection methods are discussed in detail below). The classification methods include our own methods BioHEL and GAssist, a support vector machine [37], a Random Forest classifier (RF) [38] and the “Prediction Analysis of Microarrays” method (PAM) [39]; see flowchart in Fig. 1.

In the last step of the protocol, we use a literature mining analysis to compare rankings of informative genetic probes (referred to as *genes* in the Results section, because all selected genetic probes could be mapped to a unique gene identifier via the mapping information provided by the chip manufacturer), obtained from classical feature selection methods and from a post-processing of the rule-based models generated by the BioHEL approach.

### Datasets

All methods are evaluated on three public microarray cancer datasets representing three different types of cancer: Prostate cancer (52 tumor samples vs. 50 controls) [40], lymphoma (58 Diffuse large B-cell lymphoma samples vs. 19 follicular lymphoma samples) [41], and a breast cancer dataset obtained from the collaborating Queens Medical Centre in Nottingham (84 luminal samples vs. 44 non-luminal samples) [6], [42]–[44] (see Table 1). Details for each dataset and pre-processing method used in this comparative evaluation are provided in the Material S1. All pre-processed datasets are also available online (http://icos.cs.nott.ac.uk/datasets/microarray.html), including the cross-validation subsets after feature selection.

**Table 1:** Datasets used in this paper.

Dataset	Platform	No. of genes	No. of samples	References
			class 1; class 2	
Lymphoma	Affymetrix	7,129	58 (D); 19 (F)	[41]
Prostate	Affymetrix	12,600	52 (T); 50 (N)	[40]
Breast	Illumina	47,293	84 (L); 44 (N)	[6], [42]–[44]

### Feature Selection Methods

The high number of features (genetic probes) and the relatively small number of observations (samples) in typical microarray studies pose various statistical problems, which are known as the “curse of dimensionality” in machine learning (see [45]). Therefore, after the normalization and pre-filtering of the original datasets, we apply different feature selection approaches to extract compact sets of discriminative attributes prior to the application of classification methods. Moreover, in order to evaluate to which extent our evolutionary machine learning approaches BioHEL and GAssist are capable of classifying samples without prior attribute selection, we evaluate the predictive performance of these approaches both with and without a dedicated external feature selection.

To account for the diversity of feature selection methods, three types of selection approaches are considered separately: A univariate filter (PLSS [46]), a combinatorial filter (CFS [36]) and an embedded selection approach (RFS [38]). Importantly, we only consider algorithms which are guaranteed to have a feasible runtime even on very large datasets, and instead of attempting to identify all relevant features, we aim at avoiding the selection of redundant features, which can degrade the classification performance (see [47] for a comparison of the *all relevant* selection problem against the *minimal-optimal* selection problem considered here). For a general review on feature selection approaches in bioinformatics, please see [48].

For all feature selection methods the maximum feature subset size was set to 30 to prevent over-fitting, reduce the model complexity and the probability of including false positive features (however, the methods are allowed to flexibly select less than 30 features). This upper bound was chosen according to the results of studies estimating the approximate number of features to be selected in different types of microarray studies to obtain only genetic probes with significant informative value on the outcome attribute (using different models to compute p-value significance scores, see [49]–[51]). The selection methods are described in detail in the following paragraphs.

### Partial-Least-Squares Based Feature Selection (PLSS)

As a representative of a classical univariate filter, a method using the Partial Least Squares (PLS) [52] algorithm is employed. Specifically, the features are ordered by the absolute values of the weight vector defining the first latent component in a PLS model that was built upon the training data. As previously shown [53], the ordering of features obtained from this approach is equivalent to the F-statistic used in analysis of variance (ANOVA). Thus, instead of the PLS calculation, the F-statistic itself could have been used, but PLSS provides a more efficient way of performing the computation (the fast SIMPLS algorithm [54] is used for this purpose).

### Correlation Based Feature Selection (CFS)

The combinatorial filter method CFS [36] searches for subsets of features that have high correlation to the outcome variable but low correlation amongst each other. This concept is formalized by the following feature subset score:
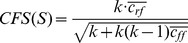
(1)where 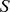 is the selected subset with 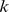 features, 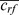 is the average feature-class correlation and 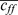 the average feature-feature correlation. While the denominator reduces the score for correlated features to eliminate redundant variables, the numerator promotes features with high correlation to the class variable to retain them as powerful discriminators. As proposed in the original CFS publication, a greedy best-first search strategy was employed to explore the feature subset space [36].

### Random Forest based Feature Selection (RFS)

In contrast to the CFS and the PLSS algorithm, the attribute selection based on the Random Forest classifier [38] uses a method directly embedded into the prediction algorithm. Specifically, a Random Forest model is built by training many binary, unpruned decision trees on bootstrap sub-samples of the training data. The importance of a feature can be evaluated based on the Gini index node impurity measure [55], by calculating the mean decrease in this measure (MDG) from parent nodes to their direct descendent nodes over all tree nodes, or alternatively, by the mean decrease in accuracy (MDA). Different machine learning studies have obtained different results regarding the comparative robustness of the MDA and MDG [56], [57], but on microarray gene expression data the results for these two impurity measures have been observed to be very similar [58]. Thus, only the MDG criterion will be considered in this study. A feature subset is obtained from the corresponding attribute ranking by selecting the top 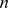 features (here, 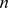 is chosen such that the obtained subset sizes are comparable to those in the CFS method).

### Classification: BioHEL and GAssist

**Figure 2:**
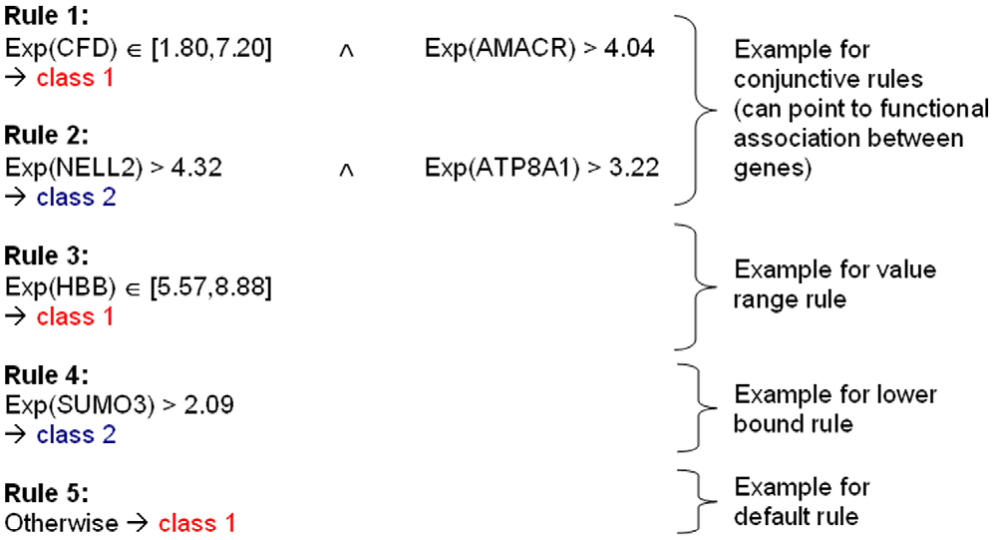
A BioHEL classification rule set obtained for the prostate cancer dataset and illustrating different types of rules. “Exp(x)” is short for “Expression of gene x”, where x is a HUGO gene symbol, “
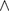
” represents the conjunctive AND-operator, “[x,y]” is an interval of expression values in which the value of the attribute must lie to fulfill one premise of the rule, and “-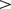
” is a class assignment operator, followed by the output class of the rule. Rule 5 is a default rule that applies if no rule above is matched.

BioHEL (Bioinformatics-Oriented Hierarchical Learning) [12]–[15] is an evolutionary machine learning system employing the Iterative Rule Learning (IRL) paradigm [59], [60] (BioHEL’s source code is available online: http://icos.cs.nott.ac.uk/software/biohel.html). The IRL procedure begins with an empty rule set and the complete set of observations as input. Classification rules are added iteratively to the set of rules until their combination covers all samples. The final outputs are structured rule sets, also known as *decision lists* [61]. A real example rule set obtained on the prostate cancer dataset is shown in Fig. 2 and highlights the different rule types in BioHEL: *Conjunctive rules*, which can provide information on potential functional associations between genes; *value range rules*, which highlight the preferential up- or down-regulation of genes under different biological conditions and the robustness for a class assignment in terms of the relative width or narrowness of an expression value range; and *default rules*, which apply if none of the previous specific rules is matched. Each time a new decision rule has been learnt and added to a corresponding rule set, the observations it covers are removed from the examples set.

To explore the search space of possible rules efficiently, BioHEL uses a standard generational Genetic Algorithm (GA) which is applied in each IRL iteration to find the best rule for samples which have not yet been covered by rules found in previous iterations. Since GAs are non-deterministic, multiple repetitions of the rule learning process with identical training sets can be used to increase the probability of finding the optimal rule. Additionally, repetitions of the complete learning process (i.e. generating a complete rule set and not just a single rule) can also be applied, in order to combine several rule sets to a majority-vote consensus prediction and benefit from the variance-reducing effects of ensemble learning [62].

In order to find the best rule in each IRL iteration, the fitness function used in the GA accounts both for the accuracy and the generality, i.e. the number of covered observations, of a rule. In BioHEL, this fitness function is based on the Minimum Description Length (MDL) principle [63] and rewards rules with.

- high accuracy, i.e. rules that classify most samples correctly,
- high coverage, i.e. rules that match many samples, and
- low complexity, i.e. rules with simple predicates.

The exact definition of BioHEL’s fitness function has been presented and discussed elsewhere [15]. However, as regards the rule coverage, it is worth mentioning that rules in BioHEL which cover a certain minimum percentage of observations receive a high reward, but after surpassing this threshold, the additional reward for covering more samples is smaller.

BioHEL has been strongly influenced by its predecessor software *GAssist* [16]–[20] (http://icos.cs.nott.ac.uk/software/gassist.html), from which it has inherited the knowledge representation. In contrast to the IRL approach employed in BioHEL, GAssist is a Pittsburgh-style learning classifier system [64], i.e. the individuals that are evolved in a generational GA are not single classification rules but rule sets representing complete tentative solutions of the data mining problem. For the exact definition of GAssist’s fitness formula, please see [16].

Previous empirical comparisons of BioHEL and GAssist have shown that GAssist tends to perform better on small datasets, whereas its successor BioHEL provides superior performance on large datasets, both in terms of number of instances and/or number of attributes. Thus, we employ both methods here to investigate their relative predictive power on microarray data. In particular, BioHEL was the only predictor for which an application on microarray data without external feature selection was possible in a feasible runtime for the LOOCV runs, hence, this learning method was applied both with and without external feature selection.

The cross-validation procedure, BioHEL and the alternative benchmark algorithms and feature selection methods have been integrated into our publicly available web-based microarray data analysis software *ArrayMining* [5].

### Evaluation Methods and Implementation Parameters

The main evaluation method used in this study is a cross-validation scheme known as *two-level external cross-validation* [35]. In an *external* cross-validation, the feature selection algorithm is applied independently to each training set generated across the cycles of the validation procedure. This approach avoids the selection bias of classical internal cross-validation, where feature selection is only performed once on the whole dataset prior to the cross-validation [65]. *Two-level* external cross-validation uses an additional nested cross-validation to optimize the parameters for the prediction algorithm using a grid search. We apply this second level of cross-validation to fit the parameters for the alternative benchmark predictors SVM, RF, and PAM.

BioHEL is used with the same default parameters as stated in [15] except for the number of iterations which is set to 500 and the probabilities for generalization and specialization which are set to 0.5. GAssist is applied using its default parameters [19] except for the number of iterations which is set to 500 as well. Both GAssist and BioHEL were run 100 times for each training set with different random seeds. Each run resulted in a rule set. An ensemble of the resulting 100 rule sets was used to predict the corresponding test set.

In order to compare BioHEL and GAssist against commonly used methods for microarray sample classification, the whole cross-validation procedure was applied to three alternative benchmark classifiers: A support vector machine (SVM) [37], a random forest classifier (RF) [38] and the “Prediction Analysis of Microarrays” method (PAM) [39].

The support vector machine we use is a linear kernel C-SVM from the e1071-package of the R statistical learning environment, a wrapper for the well-known LibSVM library. Other polynomial kernels and the radial basis function kernel were tested without providing superior results in our experiments (data not shown). This observation matches well to earlier findings in the literature according to which linear kernel SVMs often perform similar or better on microarray data than SVMs using polynomial kernels of higher degree [66], [67]. To employ the RF and PAM method, we used the corresponding R packages *randomForest* and *pamr* which are both available on the website of the Comprehensive R Archive Network (CRAN, http://cran.r-project.org).

For the comparison of our method with alternatives from the literature we only considered approaches using cross-validation for evaluation, since methods based on a single random training/test set partition are now widely regarded as unreliable [65]. For the same reason, we also exclude methods from the literature using internal cross-validation instead of external cross-validation, wherever this was clearly stated by the authors.

Since higher-level statistical analysis of microarray data can depend significantly on the data pre-processing procedure, we additionally investigate the robustness of the prediction and feature selection results for different pre-processings applied to the largest benchmark dataset. New pre-processings were obtained by using two different fold-change filters and 4 different settings for the maximum number of selected features, and the entire analytical protocol was run again for each of these variants. The stability of the results was analyzed both in terms of the cross-validated prediction results and the number of shared selected features across all the CV-cycles (see Material S1 for the results and discussion of all robustness analyses).

Importantly, the obtained prediction models are only applicable to samples from the same platform, cell type, environmental conditions and experimental procedure. However, as our classifiers support both continuous and discretized input data, they are compatible with most of the cross-study normalization methods that have been proposed in the literature to extend the applicability of machine learning models across different experimental platforms (we have previously developed a corresponding software framework that provides access to several of these cross-platform integration methods online [5]).

### Literature Mining Analysis of Selected Genes

The statistically significant differential expression of genes and their utility as predictors in a machine learning model for sample classification can indicate functional associations between these genes and the biological conditions of the cells under consideration (strictly speaking, our models use genetic probes instead of genes, but since we obtained a unique mapping for all selected probes, we will refer to their corresponding genes in the following). However, although these information sources are useful for the prioritization of candidate disease genes in biomedical studies, only experimental evidence or previous knowledge from the literature can demonstrate a functional association with the biological conditions of interest.

One of the most promising candidate genes obtained from our analysis of the breast cancer dataset was successfully evaluated in an experimental study in collaboration with the Queen’s Medical Centre in Nottingham by immunohistochemistry using tissue microarrays across 1140 invasive breast cancer samples (see our previous publication [6], the visualization of the dataset in [68], and the Results section below), however, an experimental validation of all top-ranked genes across all three microarray cancer datasets was not within the scope of this study.

Therefore, in order to examine potential associations between the disease conditions represented by the three datasets and the informative genes obtained from the feature selection methods and the most frequently occurring attributes in BioHEL’s rule sets, a literature mining analysis was applied to these genes using full-text articles from the PubMed database. Specifically, we scored putative associations between standardized names of top-ranked genes and disease terms from a controlled vocabulary (the Medical Subject Headings (MeSH) disease headings) by determining the frequency of occurrence and co-occurrence of the corresponding terms and computing the pointwise mutual information (PMI) [69]. The PMI of two terms 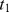 and 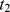, occurring with relative frequency f(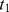) and f(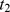), and co-occurring with relative frequency f(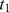,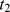) in a database of documents is defined as follows:
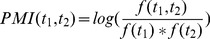
(2)


The specific MeSH disease terms used here were “prostatic neoplasms” for the prostate cancer dataset, “breast neoplasms” for the breast cancer dataset, and “lymphoma, b-cell” for the b-cell lymphoma dataset (PubMed articles are manually annotated by experts with these and other terms from the MeSH controlled vocabulary thesaurus). The PMI-value for a pair of gene/disease terms can thus be used to rank and prioritize potential functional associations, and similar PMI-based scoring schemes have previously been used to rank the similarity of genes and drugs using literature mining [70].

Since the PMI-scores for single gene/disease term pairs are not reliable enough to compare the utility of different disease gene prioritizations, we first computed the sum of positive PMI-scores across all top-ranked genes obtained from either the feature selection methods or the most frequently occurring attributes in the BioHEL rules sets. Genes with negative PMI-scores were considered as irrelevant and the corresponding score was set to zero, since the magnitude of negative scores is likely subject to random noise. The final sums of scores were compared against corresponding scores for 100 randomly selected matched-size gene sets from the corresponding microarray platforms. P-value significance scores were estimated by the proportion of times higher PMI-scores were achieved by the random model in comparison to the algorithmic selection methods. The top-ranked genes were defined as those genes that had been selected by at least two different feature selection methods, (i.e. genes corresponding to an ensemble selection), which resulted in compact sets of less than 20 selected attributes for each of the three datasets (see Results section). The same numbers of genes were selected from the most frequently occurring features in the BioHEL rules sets in order to obtain a fair comparison between this BioHEL-based feature selection and the ensemble feature selection obtained from the dedicated selection methods.

## Results and Discussion

### Comparison of Prediction Results

An overview of the comparative prediction results obtained with all combinations of feature selection, prediction methods and datasets is given in Table 2 for 10-fold CV and Table 3 for LOOCV. Below the results for all datasets are discussed.

**Table 2:** 10-fold cross-validation results.

Dataset	Feature Selection	Classification	AVG (%)	STDDEV
	CFS	BioHEL	91	8
	CFS	GAssist	93	10
	CFS	SVM	90	10
	CFS	RF	92	11
	CFS	PAM	91	10
	PLSS	BioHEL	92	8
	PLSS	GAssist	93	9
Prostate cancer	PLSS	SVM	90	11
	PLSS	RF	92	9
	**PLSS**	**PAM**	**94**	**8**
	RFS	BioHEL	89	8
	RFS	GAssist	92	12
	RFS	SVM	88	8
	RFS	RF	93	9
	RFS	PAM	90	11
	**none**	**BioHEL**	**94**	**8**
	CFS	BioHEL	81	10
	CFS	GAssist	80	15
	CFS	SVM	87	12
	CFS	RF	87	16
	CFS	PAM	78	17
	PLSS	BioHEL	93	12
Diffuse large	PLSS	GAssist	94	6
B-cell lymphoma	PLSS	SVM	91	13
	PLSS	RF	87	8
	PLSS	PAM	86	11
	RFS	BioHEL	91	11
	RFS	GAssist	89	13
	RFS	SVM	91	13
	RFS	RF	89	13
	RFS	PAM	86	14
	**none**	**BioHEL**	**95**	**8**
	CFS	BioHEL	84	11
	CFS	GAssist	87	8
	CFS	SVM	86	9
	CFS	RF	86	7
	**CFS**	**PAM**	**89**	**7**
	PLSS	BioHEL	84	7
	PLSS	GAssist	85	5
Breast cancer	PLSS	SVM	84	7
	**PLSS**	**RF**	**89**	**5**
	PLSS	PAM	88	7
	RFS	BioHEL	86	5
	RFS	GAssist	88	6
	RFS	SVM	80	17
	**RFS**	**RF**	**89**	**5**
	RFS	PAM	88	7
	**none**	**BioHEL**	**88**	**5**

10-fold cross-validation results obtained with BioHEL, SVM, RF and PAM on the three microarray datasets using three feature selection methods (CFS, PLSS, RFS); AVG  =  average accuracy, STDDEV  =  standard deviation; the highest accuracies achieved with BioHEL and the best alternative method are both shown in bold type for each dataset.

**Table 3:** Leave-one-out cross-validation results.

Dataset	Feature Selection	Classification	AVG (%)	STDDEV
	CFS	BioHEL	92	27
	CFS	GAssist	93	25
	CFS	SVM	89	31
	**CFS**	**RF**	**95**	**22**
	CFS	PAM	90	30
	**PLSS**	**BioHEL**	**94**	**24**
	PLSS	GAssist	92	27
Prostate cancer	PLSS	SVM	93	25
	PLSS	RF	93	25
	PLSS	PAM	93	25
	RFS	BioHEL	88	32
	RFS	GAssist	93	25
	RFS	SVM	89	31
	RFS	RF	91	29
	RFS	PAM	91	29
	none	BioHEL	92	27
	CFS	BioHEL	84	36
	CFS	GAssist	87	34
	CFS	SVM	88	32
	CFS	RF	87	34
	CFS	PAM	84	37
	PLSS	BioHEL	92	26
Diffuse large	PLSS	GAssist	92	27
B-cell lymphoma	**PLSS**	**SVM**	**94**	**25**
	PLSS	RF	90	31
	PLSS	PAM	86	35
	RFS	BioHEL	88	32
	RFS	GAssist	88	32
	RFS	SVM	90	31
	RFS	RF	92	27
	RFS	PAM	83	38
	**none**	**BioHEL**	**94**	**25**
	CFS	BioHEL	82	38
	CFS	GAssist	84	36
	CFS	SVM	84	37
	CFS	RF	84	36
	**CFS**	**PAM**	**90**	**30**
	PLSS	BioHEL	84	37
	PLSS	GAssist	84	36
Breast cancer	PLSS	SVM	81	39
	PLSS	RF	88	33
	PLSS	PAM	86	35
	RFS	BioHEL	82	39
	RFS	GAssist	85	36
	RFS	SVM	86	35
	RFS	RF	87	34
	RFS	PAM	88	32
	**none**	**BioHEL**	**86**	**35**

Leave-one-out cross-validation results obtained with BioHEL, SVM, RF and PAM on the three microarray datasets using three feature selection methods (CFS, PLSS, RFS); AVG  =  average accuracy, STDDEV  =  standard deviation; the highest accuracies achieved with BioHEL and the best alternative are both shown in bold type for each dataset.

### Prostate Cancer

On the prostate cancer dataset, the best prediction results with BioHEL were reached without external feature selection, providing an average accuracy of 94% (10-fold CV), or when combining BioHEL with the PLSS filter (avg. acc. 94%, LOOCV). Among the alternative benchmark classifiers considered in this study (SVM, RF and PAM, see Tables 2 and 3) only the PLS/PAM combination achieved the same accuracy for 10-fold CV and the CFS/RF combination reached a slightly higher accuracy for LOOCV (95%). GAssist reached accuracies of at least 92% in combination with all feature selection methods, but was outperformed by the best BioHEL models.

Similarly, in the cross-validation results reported in the literature for this dataset only the methods by Shen et al. (94.6%) [71] and Paul et al. (96.6%) [72] (see Table 4) obtained a similar or slightly higher average accuracy than BioHEL. For comparison, Shen et al. employ a singular value decomposition (SVD) instead of feature selection, which includes more genes from the original data than the maximum of 30 considered here for all feature selection methods. This type of model can be more difficult to interpret than decision rule models without feature transformation (unless the derived features can be linked to biological processes). Paul et al. use the original features in their models, but the average number of included genes also exceeds 30 features (48.5). Thus, in comparison to state-of-the-art benchmark classifiers and alternative approaches in the literature, BioHEL reaches similar levels of accuracy, in spite of the simple nature of its rule-base models, which facilitates the biological interpretation for the experimenter.

**Table 4:** Comparison of prediction results from the literature for the prostate cancer dataset.

Author (year)	Method	AVG (%)	Size
T.K. Paul *et al.* [72]	RPMBGA, LOOCV	96.6	48.5
Wessels *et al.* [73]	RFLD(0), Monte-Carlo CV	93.4	14
Shen *et al.* [71]	PLR, Monte-Carlo-CV (30 iterations)	94.6	***
	LSR, Monte-Carlo-CV (30 iterations)	94.3	***
W Chu *et al.* [124]	Gaussian processes, LOOCV	91.2	13
Lecocke *et al.* [125]	SVM, LOOCV	90.1	**
	DLDA, LOOCV	89.2	**
	GAGA+3NN, LOOCV	88.1	**
our study	BioHEL, 10-fold CV	94	*30
	PLSS+BioHEL, LOOCV	94	*30

(*maximum no. of genes per base classifier in ensemble learning model; **evaluation results averaged over feature subsets using different numbers of genes; ***singular value decomposition used instead of classical feature selection).

### Diffuse Large B-cell Lymphoma

On the DLBCL dataset, the highest average sample classification accuracies of 95% (10-fold CV) and 94% (LOOCV) were both obtained when using BioHEL without any feature selection. Moreover, none of the parameter-optimized benchmark methods reached higher accuracies, only the PLSS/SVM combination provided the same accuracy as BioHEL. In the results reported in the literature for this dataset (see Table 5), only the approach by Wessels et al. [73] reached a slightly higher accuracy (96%) than the best BioHEL models, but using a feature subset size of 80 genes, almost 3 times more than the maximum number of features allowed into BioHel’s rules. GAssist provided accuracies in a similar range of values as the other benchmark methods and reached its best results (10-fold CV: 94%, LOOCV: 92%) in combination with the PLSS filter.

**Table 5:** Comparison of prediction results from the literature for the lymphoma dataset.

Author (year)	Method	AVG (%)	Size
Wessels *et al.* [73]	RFLD(10), Monte-Carlo CV	95.7	80
Liu *et al.* [126]	MOEA+WV	93.5	6
Shipp *et al.* [41]	SNR+WV, LOOCV	92.2	30
Goh *et al.* [127]	PCC-SNR + ECF, LOOCV	91	10
Lecocke *et al.* [125]	GA+SVM, LOOCV	90.2	**
	GAGA+DLDA, LOOCV	89.8	**
	GAGA+3-NN, LOOCV	86.3	**
Hu *et al.* [128]	WWKNN, LOOCV	87.01	12
	ECF, LOOCV	85.71	12
our study	BioHEL, 10-fold CV	95	*30
	BioHEL, LOOCV	94	*30

(*maximum no. of genes per base classifier in ensemble learning model; **evaluation results averaged over feature subsets using different numbers of genes).

A common problem in the classification of high-dimensional data with small sample sizes is the high variance in cross-validation error estimates, especially in LOOCV [65], [74]. This observation was also made on the three datasets considered in this study and applies both to BioHEL and the alternative prediction methods. Thus, in spite of the high average accuracy reached by some method combinations, the lack of robustness still hinders the use of these approaches in routine clinical application.

### Breast Cancer

For the breast cancer dataset, obtained from the Nottingham Queen’s Medical Centre, the best average accuracies with BioHEL were again obtained when using no external feature selection, providing average accuracies of 88% (10-fold CV) and 86% (LOOCV). These results were similar to those of other benchmark classifiers, with some methods being slightly superior and some slightly inferior (the most successful approach was CFS/PAM with 89% acc. for 10-fold CV and 90% acc. for LOOCV). Importantly, independent of the feature selection and cross-validation method, BioHEL always provided average accuracies of at least 82% on the breast cancer data. GAssist again did not reach BioHEL’s best performance, but provided robust accuracies of at least 84% across all feature selection methods.

The lower accuracies achieved by all methods on the breast cancer data in comparison to the performances of these methods observed on the other datasets matches to previous observations showing that the classification of breast cancer microarray samples tends to be more difficult than the discrimination of gene array samples for other cancer types [75]. Since the breast cancer dataset considered here was obtained from a collaborating institute, no external cross-validation results for alternative methods are available in the literature, however, the dataset has been published online (http://www.ebi.ac.uk/microarray-as/ae/browse.html?keywords=E-TABM-576) and can freely be used for comparative evaluation and biological analysis purposes.

On the whole, on all three datasets the BioHEL classification models provided classification accuracies that were among the highest in comparison to current benchmark classification methods and other approaches from the literature. The similarity between the averaged accuracies obtained from 10-fold CV and LOOCV shows that these performance estimates are stable and do not vary significantly with the chosen validation scheme (regarding the variance of the accuracy across the cross-validation cycles, as expected, lower variances are obtained when using the larger test set sizes in 10-fold CV). Similarly, the comparison of prediction and feature selection results across different dataset pre-processings suggests that BioHEL’s performance is robust across a wide range of pre-filtering settings (see Material S1 for detailed results and discussion of the robustness analyses).

In order to objectively compare the classifiers across all datasets and different feature selection methods, we additionally applied a Friedman test [76], [77] over the average classification accuracies across all feature selection methods (once for 10-fold CV and once for LOOCV). According to this statistical test, GAssist and the RF method performed best for 10-fold CV and obtained the same average rank (see Table 6) when using feature selection. Interestingly, BioHEL obtained lower average ranks than these methods when being combined with external feature selection, but provided the overall highest accuracies without external attribute selection. This result suggests that BioHEL exploits the information content of features that are considered as insignificant by the dedicated selection methods and thus avoids a performance bottleneck resulting from the prior application of an external selection approach.

**Table 6:** Comparison of prediction methods.

	Average ranks				
method	SVM	RF	PAM	BioHEL	GAssist
10-fold	3.8	**2.3**	3.1	3.4*	**2.3**
LOO	3.0	**2.2**	3.1	3.7*	3.0

Results of a Friedman test to compare prediction methods across different datasets and feature selection methods (the best average ranks are shown in bold typeface; *here only the results in combination with feature selection are taken into account).

For LOOCV, similar results were obtained as with 10-fold CV, with the exception that RF achieved a better average rank than GAssist with the second best rank. Again, BioHEL tends to obtain lower average ranks in combination with external attribute selection than the other approaches, but achieves the best overall performance without external selection procedure.

Overall these results show that both systems (GAssist in combination with feature selection and BioHEL without external selection) not only generate compact and easy-to-interpret decision rules but also achieve the best or close-to-best performance in all tested scenarios. Thus, BioHEL and GAssist enable experimenters to benefit from the enhanced interpretability of a rule-based learning approach without having to sacrifice performance in comparison to other state-of-the-art approaches.

Across all methods, the overall memory requirements and the runtimes for applying the trained models were similar and negligibly small on a standard desktop machine (single applications of an algorithm required a few minutes or less on a 2 GHz CPU). The most time-consuming cross-validation experiment (combining LOOCV with the 100-times BioHEL ensemble and repeating this 10 times for different random seeds) required less than one day.

### Comparison of Feature Selection Results

When using feature selection prior to supervised classification, the average accuracy often varies greatly with the choice of the selection method, since the predictive performance does not only depend on the inclusion of informative features, but can also be affected negatively by the selection of redundant and irrelevant features. To compare the three selection methods considered in this study (CFS, RFS, PLSS), the Friedman test was applied to the average classification accuracies (once for 10-fold CV and once for LOOCV) across all datasets and all five prediction methods (BioHEL, GAssist, SVM, RF and PAM).

In summary, according to a Holm-test applied after the Friedman test, PLSS was significantly superior to CFS both for 10-fold CV (confidence level: 80%) and LOOCV (confidence level: 90%), and also superior to RF for LOOCV (confidence level: 80%, no other significant differences were detected above this confidence level). Similarly, PLSS obtained the best average ranks in the Friedman test for both 10-fold CV and LOOCV (see Table 7). These observations, showing that a univariate ranking method outperformed a combinatorial filter and an embedded selection method, could indicate that the feature independence assumption behind the univariate approach is reasonably satisfied for the most informative features, and justify the widespread popularity of univariate approaches in microarray gene selection. Relatively high performances of univariate selection strategies had already been noted in a similar study on microarray data by Wessels et al. [73], comparing other algorithms. However, an alternative interpretation of these results might be that feature dependencies detected by the multivariate selection methods were false positives, resulting in a weaker predictive performance of these approaches. Thus, if the independence assumption represents a good approximation for some of the most informative features, or if multivariate methods fail to correctly capture the dependence structure between different variables, a fast univariate selection approach can be the method of choice for complex, high-dimensional microarray data.

**Table 7:** Comparison of feature selection methods.

	Average ranks		
method	CFS	PLSS	RFS
10-fold	2.3	**1.8**	2.0
LOO	2.3	**1.6**	2.0

Results of a Friedman test to compare feature selection methods in terms of classification accuracy across different datasets and prediction methods (the best average ranks for each row are shown in bold typeface).

### Literature Mining Analysis of Selected Genes - Results

To illustrate the utility of the analysis pipeline for biological interpretation of the data and to compare the ensemble of the external feature selection methods with BioHEL’s capacity to directly identify informative features during the model generation, an example literature mining was performed for the top-ranked genes on each dataset (see Methods section). When considering only genes chosen by at least two different selection methods among the genes selected most frequently across the LOOCV cycles, 11 genes were obtained for the prostate cancer dataset (see Table 8 left), 10 genes for the lymphoma dataset (Table 9 left) and 18 genes for the breast cancer dataset (Table 10 left). To compare these selection results against a ranking of genes according to the frequency of their occurrence in the rule sets generated by the BioHEL 100-times ensemble across all LOOCV cycles, the same numbers of genes were selected from the top of these rankings for each datasets (see Tables 8, 9 and 10, right side, the shared genes detected as informative by both approaches are highlighted in bold face).

**Table 8:** List of high scoring genes for the prostate cancer dataset.

Ensemble feature selection			BioHEL feature ranking		
Gene identifier	Freq.	Annotation	Gene identifier	Perc.	Annotation
**37639_at**	3	*hepsin (transmembrane protease, serine 1)*	**32598_at**	7.6	*nel-like 2*
**32598_at**	3	*nel-like 2*	914_g_at	4.0	*transcriptional regulator ERG*
**41706_at**	3	*alpha-methylacyl-coa racemase (AMACR)*	**37639_at**	3.4	*hepsin (transmembrane protease, serine 1)*
38634_at	3	*retinol binding protein 1, cellular (CRBP1)*	**40282_s_at**	2.0	*complement factor d (adipsin)*
37366_at	3	*pdz and lim domain 5 (PDLIM5)*	41817_g_at	1.5	*caspase recruitment domain family, member 10*
**40282_s_at**	2	*complement factor d (adipsin)*	35278_at	1.5	*ribosomal protein S29*
38087_s_at	2	*s100 calcium binding protein a4 (S100A4)*	41741_at	1.3	*RNA-binding motif protein 3*
41468_at	2	*T cell receptor gamma (TCR-gamma)*	32250_at	1.3	*complement factor H*
38827_at	2	*anterior gradient 2 (AGR2)*	32755_at	1.1	*actin, alpha 2, smooth muscle, aorta*
38406_f_at	2	*prostaglandin d2 synthase 21kda (PTGDS)*	**41706_at**	1.0	*alpha-methylacyl-coa racemase (AMACR)*
34840_at	2	*we38g03.x1 homo sapiens cdna, 3′ end*	37331_g_at	0.9	*aldehyde dehydrogenase 4 family, member A1*

List of genes that were chosen by at least two different selection methods among the 20 features selected most frequently on the prostate dataset. The 4 genes detected as informative by both the Ensembl FS and the BioHEL FR approach (*hepsin*, *nel-like 2*, *AMACR* and *adipsin*) are highlighted in bold face (see discussion in the literature mining analysis section).

**Table 9:** List of high scoring genes for the lymphoma dataset.

Ensemble feature selection			BioHEL feature ranking		
Gene identifier	Freq.	Annotation	Gene identifier	Perc.	Annotation
X02152_at	3	*lactate dehydrogenase a (LDHA)*	X01060_at	6.6	*Transferrin receptor protein 1*
V00594_at	2	*metallothionein 2a (MT2A)*	M63835_at	6.0	*Immunoglobulin Gamma Fc receptor I*
HG1980-HT2023_at	2	*tubulin, beta 2c (TUBB2C)*	HG2090-HT2152_s_at	5.3	*CD163 molecule*
U63743_at	2	*kinesin family member 2c (KIF2C)*	X02544_at	3.0	*orosomucoid 1*
X05360_at	2	*cell division cycle 2, g1 to s and g2 to m (CDC2)*	U21931_at	1.9	*fructose-1,6-bisphosphatase 1*
M63379_at	2	*clusterin*	D80008_at	1.7	*GINS complex subunit 1 (Psf1 homolog)*
M13792_at	2	*adenosine deaminase (ADA)*	X65965_s_at	1.5	*superoxide dismutase 2, mitochondrial*
L19686_rna1_at	2	*macrophage migration inhibitory factor (MIF)*	D13413_rna1_s_at	1.3	*solute carrier family 36, member 2*
D14662_at	2	*peroxiredoxin 6 (PRDX6)*	L25876_at	1.2	*cyclin-dependent kinase inhibitor 3*
S73591_at	2	*thioredoxin interacting protein (TXNIP)*	D78134_at	1.1	*cold inducible RNA binding protein*

List of genes that were chosen by at least two different selection methods among the 30 features selected most frequently on the lymphoma dataset. On this dataset, the genes detected as informative by the Ensembl FS and the BioHEL FR did not overlap (see discussion in the literature mining analysis section).

**Table 10:** List of high scoring genes for the breast cancer dataset.

Ensemble feature selection			BioHEL feature ranking		
Gene identifier	Freq.	Annotation	Gene identifier	Perc.	Annotation
**GI_4503602-S**	3	*estrogen receptor 1 (ESR1)*	GI_37545993-S	0.7	*Serpin A11 precursor*
**GI_14249703-S**	3	*RAS-like, estrogen-regulated, growth-inhibitor* *(RERG)*	**GI_14249703-S**	0.6	*RAS-like, estrogen-regulated, growth-inhibitor (RERG)*
**GI_16507967-S**	3	*potassium channel, subfamily K, member 15 (KCNK15)*			
**GI_22779933-S**	2	*WD repeat membrane protein PWDMP (PWDMP)*	GI_23308560-S	0.6	*RNA-binding protein 24*
GI_42657473-S	2	*Uncharacterized protein (C6orf115)*	**GI_22779933-S**	0.6	*WD repeat membrane protein PWDMP (PWDMP)*
GI_7706686-S	2	*Enah/Vasp-like (EVL)*	**GI_16507967-S**	0.5	*potassium channel, subfamily K, member 15 (KCNK15)*
GI_40788002-S	2	*proteasome (prosome, macropain)*	GI_22748948-S	0.4	*IGF1R protein*
		*activator subunit 4 (PSME4)*			
GI_33620752-S	2	*hypothetical protein FLJ10876 (FLJ10876)*	GI_4507266-S	0.4	*Stanniocalcin-2 precursor (STC-2)*
**GI_13236596-S**	2	*DDB1 and CUL4 associated factor 10 (WDR32)*	**GI_29029609-A**	0.4	*pyrimidinergic receptor P2Y6,* *G-protein coupled*
**GI_29029609-A**	2	*pyrimidinergic receptor P2Y6, G-protein coupled*	GI_4502798-S	0.4	*Chondroadherin precursor*
GI_37551139-S	2	*hypothetical protein PRO2013 (PRO2013)*	Hs.501130-S	0.4	*GDNF family receptor alpha 1 isoform b*
GI_40255152-S	2	*potassium channel tetramerisation*	**GI_13236596-S**	0.4	*DDB1 and CUL4 associated factor 10 (WDR32)*
		*domain containing 6 (KCTD6)*	**GI_4503602-S**	0.3	*estrogen receptor 1 (ESR1)*
GI_30410031-S	2	*prostate-specific membrane antigen-like*	GI_42659577-S	0.3	*uncharacterized protein KIAA1377*
		*protein (PSMAL/GCP III)*			
GI_4503928-S	2	*GATA binding protein 3 (GATA3), mRNA*	**GI_29738585-S**	0.3	*GDNF family receptor alpha 1 (GFRA1, LOC143381)*
					*transcript variant 1*
GI_42659459-S	2	*hypothetical gene supported by AK128810 (LOC399717)*	GI_21389370-S	0.3	*ankyrin repeat domain 22*
**GI_29738585-S**	2	*GDNF family receptor alpha 1 (GFRA1, LOC143381)*	Hs.202515-S	0.3	*calcium channel, voltage-dependent, L type (CACNA1D)*
		*transcript variant 1*			
GI_38455428-S	2	*breast cancer membrane protein 11 (BCMP11),* *mRNA*	GI_18152766-S	0.3	*synaptotagmin-like 4* *(granuphilin-a) (SYTL4)*
GI_22035691-A	2	*GDNF family receptor alpha 1 (GFRA1)*	Hs.499414-S	0.3	*Cluster 499414 (chr 10), unknown function*
		*transcript variant 2*			

List of genes that were chosen by at least two different selection methods among the 30 features selected most frequently on the breast cancer dataset. The 7 genes detected as informative by both the Ensembl FS and the BioHEL FR approach are highlighted in bold face (see discussion in the literature mining analysis section).

**Figure 3:**
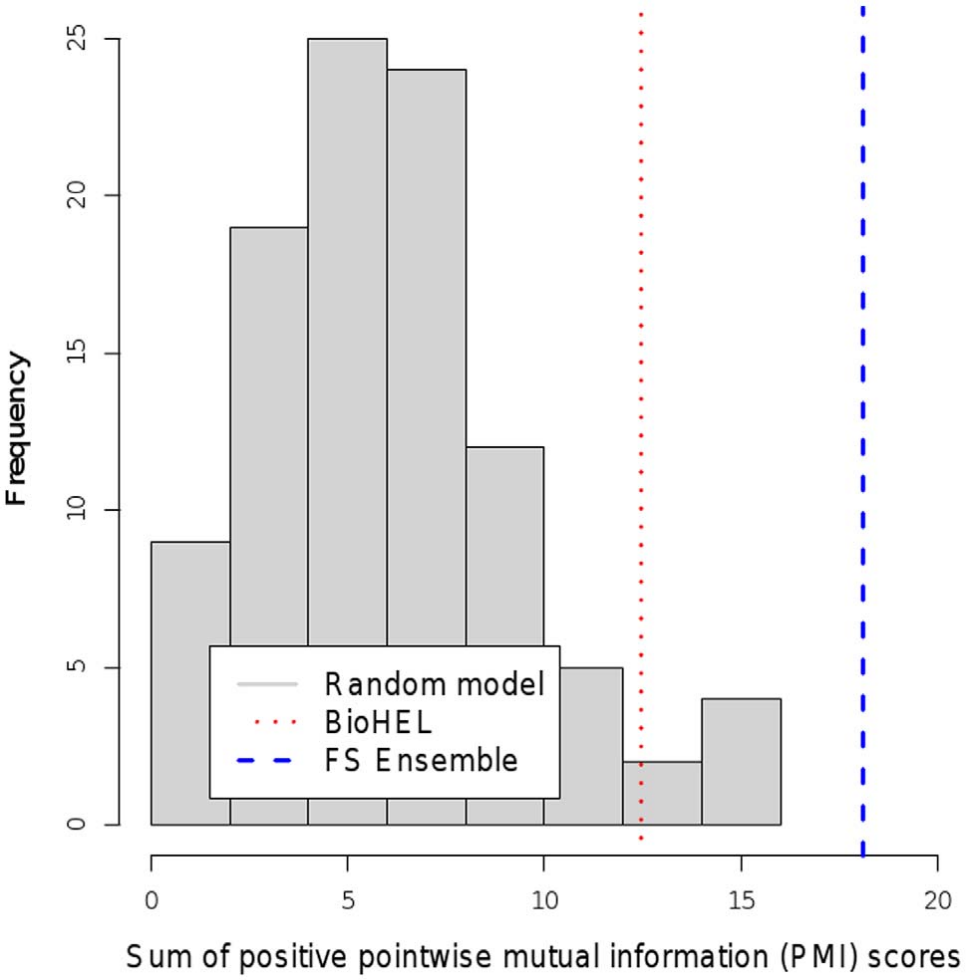
Comparison of text mining scores. Histogram of text mining scores for randomly chosen gene identifier subsets compared to scores achieved by BioHEL and the ensemble feature selection (FS) approach (prostate cancer dataset).

**Figure 4:**
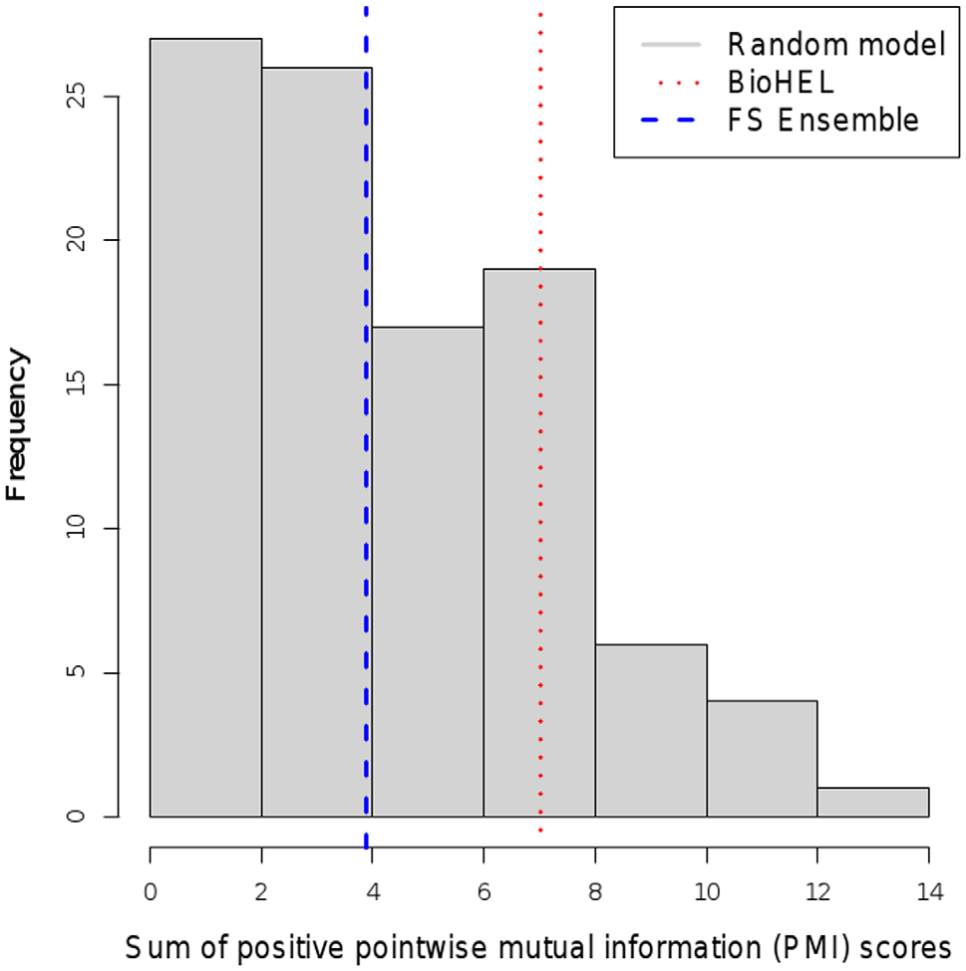
Comparison of text mining scores. Histogram of text mining scores for randomly chosen gene identifier subsets compared to scores achieved by BioHEL and the ensemble feature selection (FS) approach (lymphoma cancer dataset).

**Figure 5:**
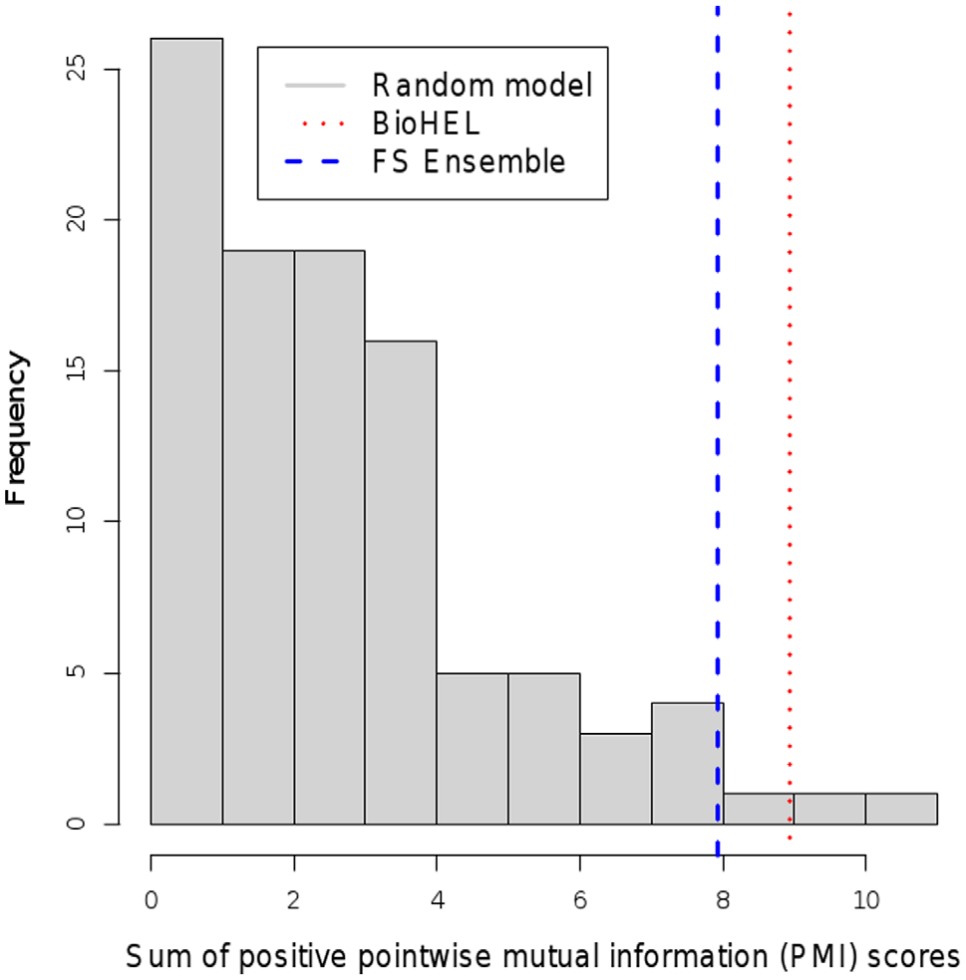
Comparison of text mining scores. Histogram of text mining scores for randomly chosen gene identifier subsets, compared to scores achieved by BioHEL and the ensemble feature selection (FS) approach (breast cancer dataset).

The two gene rankings (ensemble feature selection and BioHEL-based feature ranking) were validated externally using literature mining in the PubMed database by computing the sum of positive PMI scores (see Methods section) between the standardized gene names from the rankings and the disease terms from a controlled vocabulary matching to the three datasets (“prostatic neoplasms”, “breast neoplasms” and “lymphoma, b-cell”). The same computation was repeated 100 times on matched-size gene sets selected randomly from the genes on the corresponding microarray platforms, and the histograms of these random model PMI scores, as well as the scores achieved by the ensemble feature selection (Ensemble FS) and BioHEL-based feature ranking (BioHEL FR) are shown in Fig. 3, 4 and 5. To quantify the relative performances of the methods, the p-value significance score estimates obtained from these PMI scores are listed in Table 11.

**Table 11:** Literature mining significance scores.

Dataset	Ensemble FS (p-value)	BioHEL FR (p-value)
Prostate	0.00	0.05
Lymphoma	0.51	0.22
Breast	0.02	0.03

The table and the plots show that both the Ensemble FS and the BioHEL FR method reached significant p-values 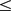 0.05 on the prostate and the breast cancer dataset; however, neither of the two approaches provided a significant p-value on the lymphoma data (BioHEL FR p-value: 0.22, Ensemble FS p-value: 0.51). The lower performance of the literature mining analysis on this dataset could result from the lower number of available samples (77 samples in relation to 102 for the prostate cancer study and 128 for the breast cancer study), the high degree of class imbalance (58 diffuse large b-cell lymphoma samples vs. 19 follicular lymphoma samples), resulting in only 10 genes that were detected as significant by at least two feature selection methods, and an overlap of zero between the top-ranked genes obtained from Ensemble FS and from BioHEL FR (see Table 9).

Regarding the relative performance of the gene selection approaches, the text-mining analysis showed that in two out of the three datasets, the lymphoma and the breast cancer dataset, BioHEL FR reached lower p-values than Ensemble FS. Accordingly, at least on some datasets, the BioHEL FR approach provides superior results in terms of the PMI literature mining scores in comparison to an ensemble of multiple selection methods, even though BioHEL FR only requires the application of a single algorithm.

Thus, the permutation-based text-mining analysis can provide insights both on the reliability of gene selection results across different datasets (in this case revealing a higher reliability obtained on the prostate and breast cancer data in comparison to the lymphoma data) and on the relative performance of the gene selection methods.

In addition to the automated text mining analysis, the functional annotations of the top-ranked genes were also investigated by manual inspection of the literature to identify specific associations with the disease conditions of interest. Since an in-depth discussion of the single genes selected by the two different approaches would exceed the scope of this study, we focus mainly on the genes that were selected by both the Ensemble FS and the BioHEL FR approach.

For the **prostate cancer dataset**, 4 genes were found in the intersection set of the two selection methods: *Hepsin*, *Neural epidermal growth factor-like 2 (nel-like 2)*, *alpha-methylacyl-coa racemase (AMACR)* and *adipsin*. Annotations for these and all other genes on the list were obtained from the Gene Cards web-service [78], the DAVID functional annotation database [79] and from the supplementary material of the microarray dataset. Canvassing of the biomedical literature reveals that all these genes have either known significant functional associations with prostate cancer, are used as diagnostic markers or have previously been proposed as new candidate markers.

Specifically, *hepsin* is a cell surface serine protease which has been found to be significantly over-expressed in prostate cancer [80]–[82] in other studies and which has also been proposed as a prognostic biomarker [83], [84]. However, there is no common agreement on the function of hepsin; while some studies claim that hepsin promotes prostate cancer progression [85], other studies suggest that hepsin inhibits cell growth in prostate cancer cells [86]. *Neural epidermal growth factor-like 2 (nel-like 2, NELL2)* is a growth factor homologue that is believed to function as a differentiation and regulation factor [87]. NELL2 has been reported to be differentially expressed in benign prostatic hyperplasia (BPH) [88], suggested as a mesenchymal regulator of organogenesis and tumorigenesis [89] and used in a patented diagnostic method for prostate cancer (US Patent App. 11/519,892). The third candidate gene, *Alpha-methylacyl-CoA racemase (AMACR)* codes for an enzyme with functional roles in bile acid biosynthesis and 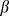-oxidation of branched-chain fatty acids [90]. It has been identified to be significantly up-regulated in prostate cancer based on different independent gene expression data sets and has also been suggested as a biomarker for prostate cancer diagnosis in various studies [84], [91], [92]. *Adipsin* is a specific gene for adipocytes [93], cells specialized in storing energy as fat which have also been suggested to affect the proliferation and differentiation of epithelial cells. Culturing of adipocytes with a prostate carcinoma cell line [94] has provided strong evidence that adipocytes modulate the growth and cytokine expression of prostate cancer cells (based on histological and immunohistochemical assays and RT-PCR measurement of cytokine expression). Though the precise role of adipsin in cancer remains unknown, its differential expression in cancer tissue has been observed in several studies [95]–[97]. However, these findings might also result from a general relationship between obesity and cancer, since obesity is a risk factor for several cancers [98], including prostate cancer, and adipsin expression is known to be impaired both in acquired and genetic obesity [99].

On the **lymphoma dataset**, no overlap was found between the top-ranked genes from the Ensemble FS and the BioHEL FR approach. As mentioned before in the section on the automatic literature mining analysis, a lower robustness of the feature selection results on this dataset might result from the lower number of available samples and the higher degree of class imbalance in relation to the other datasets. However, when inspecting the annotations for the top 3 genes on both ranking lists, again strong functional associations with the disease condition were found.

*Lactate dehydrogenase A (LDHA)*, the only gene that was detected as significant by all 3 selection methods in the Ensemble FS approach, is known to have elevated expression levels after tissue breakdown. For this reason, LDHA is already an established marker to monitor cancer patients. Moreover, a relationship between LDHA levels and the histological type and the tumor mass of non-Hodgkin’s lymphomas has been investigated in the literature [100]. *Metallothionein 2a (MT2A)*, a gene that was selected by 2 different methods in the Ensemble FS approach, codes for a protein of the metallothionin family, which is involved in many pathophysiological processes including protection against oxidative damage, cell proliferation, drug and chemotherapy resistance and cancer development [101]. Moreover, it has been shown that malignant lymphoblasts of diffuse large B-cell lymphoma have high metallothionein expression [102]. Similarly, tubulins like *TUBB2C* are known to have higher expression levels in proliferating cancer cells due to the microtubule formation in these cells. These proteins are therefore often considered as targets for anticancer drugs to inhibit the growth of cancer cells and there expression levels have also been reported to be down-regulated in treatments that induce apoptosis in lymphoma cells [103].

*Transferrin receptor protein (TfR) 1*, the top-ranked gene according to the BioHEL FR approach, is required for the import of iron into a cell by means of a receptor-mediated endocytic transport of a transferrin-iron complex, but is also involved in the regulation of cell growth. Elevated levels of TfR have been found in several malignancies, and the membrane protein has been studied as a promising target for the treatment of cancer using antibodies [104]. Moreover, the expression of TfRs has previously been reported to be correlated with survival and histological grading of non-Hodgkin’s malignant lymphoma patients [105]–[107]. *Immunoglobulin gamma Fc receptor I (FCGR1A)* is a receptor protein for immunoglobulin antibodies, which are produced by the innate immune system in response to viral and bacterial infections or cancer cells. The gene is known to be expressed as part of the immune response signature of follicular lymphoma [108] and known to be part of a cluster of over-expressed immune system related genes in B-cell lymphoma samples which are rich in T-cells and histiocytes (immune cells capable of digesting foreign substances) [109]. Interestingly, an approved drug against non-Hodgkin lymphoma, Tositumomab, is known to bind to FGR1A, although the intended target is the B-lymphocyte antigen CD20.

The *hemoglobin scavenger receptor (Cluster of Differentiation 163, CD163)* protein, encoded by the gene ranked third by BioHEL FR, is a receptor involved in clearance and endocytosis of hemoglobin/haptoglobin complexes by macrophages. CD163 has been proposed as a marker for tumour-infiltrating macrophages in Hodgkin’s lymphoma, where high CD163 expression has been observed to correlate with adverse outcome [110]. Moreover, it has been suggested that CD163 might have an anti-inflammatory role [111] and could have diagnostic value for monitoring macrophage activation in inflammatory conditions, which are considered to play a critical role in the tumour progression of many cancers [112].

For the **breast cancer data**, 7 genes were detected as informative by both the Ensemble FS and the BioHEL FR approach. We have recently evaluated one of these genes, *RAS-like, estrogen-regulated, growth-inhibitor (RERG)*, experimentally in a collaborative study with the Queen’s Medical Centre in Nottingham by immunohistochemistry using tissue microarrays across 1140 invasive breast cancer samples [6]. The study confirmed that the expression of RERG provides a sensitive marker for the discrimination between the clinically relevant categories of luminal and non-luminal breast cancer samples (see the approach by Nielsen et al. for breast cancer subtype categorisation [113]). Moreover, several significant correlations with already existing markers of luminal differentiation were identified, including positive correlations with the expression of the estrogen receptor, luminal cytokeratins (CK19, CK18 and CK7/8), FOXA1 (p-value = 0.004), androgen receptor, nuclear BRCA1, FHIT and cell cycle inhibitors p27 and p21, and inverse associations with the proliferation marker MIB1 (p-value = 0.005) and p53. More importantly, strong RERG expression showed an association with a longer breast cancer specific survival and distant metastasis free interval in the whole patient cohort and these associations were independent of other prognostic variables. These results match to the high rankings this gene received by the computational gene selection methods considered here. In the BioHEL FR approach, RERG was ranked second, in the Ensemble FS approach it belonged to the 3 only genes that were chosen as informative by all three input selection methods (see Table 10).

A further gene with high ranks in both selection approaches is *estrogen receptor 1 (ESR1)*, a well-known breast cancer marker gene, which encodes the estrogen receptor alpha (ER-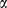). In luminal breast cancer samples, ER-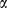 is known to be expressed in the tumour cells (ER+ type), whereas it is not expressed in basal-like samples (ER- type). The oestrogen hormone is well-known to cause the growth of ER+ breast cancer cells, and some hormone therapies are based on using anti-oestrogens as drugs against corresponding forms of breast cancer.

A third top-ranked gene, *potassium channel, subfamily K, member 15 (KCNK15, TASK-5)*, is a less obvious candidate gene and currently unknown in oncogenesis. However, KCNK15 has been found to be silenced by hypermethylation of the promotor region in many tumours [114]. The gene encodes a two-pore potassium channel protein, which corresponds to findings for other ion channels, like the Ca2^+^ channel CACNA1G and the Na^+^ channel SLC5A8 with putative tumour suppressive function, which have already been reported to be hypermethylated in different cancers [115], [116]. Thus this gene/protein might be a promising target for future investigations.

Another membrane protein selected by both approaches is the *WD repeat membrane protein (PWDMP, WDR19)*. WD-repeat proteins form a family of structurally related proteins that participate in several cellular functions, including vesicle formation, vesicular trafficking, transmembrane signaling and mRNA modification [117]. In a previous breast cancer study, WDR19 has been identified to be differentially expressed between carcinoma cells with similar morphology to healthy cells and cells with deviating, irregular morphology [118]. However, so far, WDR19 has only been implicated in other cancer types, in particular prostate cancer [119]. Interestingly, a further WD repeat-containing protein was selected by both approaches, *WDR32 (WD repeat-containing protein 32)*, also known as *DDB1 and CUL4 associated factor 10*. WDR32 has been found to be differentially expressed in breast cancer cells in several studies, as reported in the Genes-to-Systems Breast Cancer (G2SBC) database [120].

The 6th gene in the intersection set of the ranking lists is *G-protein coupled pyrimidinergic receptor P2Y6*, belonging to the group of P2Y receptors that respond to purine and pyrimidine nucleotides. P2Y6 expression has been shown to be deregulated in cases of altered progestin responsiveness due to changes in the expression of progesterone receptor isoforms, which are known to occur in breast cancer cells [121]. Moreover, in 2003 a patent application has been filed for a method to detect pre-neoplastic and neoplastic states based on P2Y expression levels (US Patent App. 10/450,205).

Finally, the gene that occurs most frequently in different transcript variants as top-ranked in both selection methods is *GDNF family receptor alpha 1 (GFRA1)*, which occurs in the transcript variants 1 and 2 and the *isoform b* version. The Glial cell line-derived neurotrophic factor (GDNF) which binds to the receptor GFRA1 is known to play an important role in differentiation and the control of neuron survival. It has also been suggested to function as a component of the inflammatory response in breast cancers, and an experimental study using a breast cancer tissue microarray has shown that *GFRA1* transcripts are over-expressed in invasive breast carcinomas, and in particular in hormone receptor–positive (ER+ and PR+) tumors [122].

In summary, all genes appearing in both lists of top-ranked genes from different selection approaches have either putative or known functional associations with the corresponding cancer type. Although this does not imply that all high-scoring genes and their corresponding products are suitable markers for the diagnosis or monitoring of cancer diseases, both the automated literature mining analysis and the manual inspection of the functional annotations of the top-ranked genes confirm the utility of these selection methods for identifying and prioritizing putative markers, and for the biological interpretation of the data.

While classical gene prioritization methods are based on a single feature selection method and a single confidence measure [123], the approaches employed here use either information from multiple selection methods or multiple prediction models given by ensembles of BioHEL rule sets. Moreover, the robustness of the selection across multiple cross-validation cycles is taken into account.

Finally, since the lists of high-scoring candidate genes which were detected by multiple feature selection methods are confined to relatively small sets of attributes, it is feasible to apply more sensitive experimental approaches to study single genes and proteins, e.g. using a quantitative polymerase chain reaction (qPCR) or immunohistochemistry on tissue microarrays, as illustrated by the successful validation of RERG gene expression as a marker for luminal-like breast cancer samples.

### Conclusion

In this paper, we have evaluated the rule-based, evolutionary machine learning systems BioHEL and GAssist for supervised microarray sample classification. Empirical results on three public microarray datasets using three feature selection methods and two external cross-validation schemes show that both methods reach comparable accuracies to current state-of-the-art prediction methods for gene array data, and achieve the best or close-to-best performance depending on the setting: BioHEL achieves the highest overall accuracies when being applied without external feature selection, whereas GAssist tends to outperform other methods when being applied in combination with feature selection. These results are corroborated by comparisons across multiple types of feature selection methods, as well as by comparisons to other methods in the literature.

As an added value, in contrast to other state-of-the-art benchmark methods, the prediction models generated by BioHEL and GAssist are based on easily interpretable *if-then-else*-rules. These benefits in terms of model interpretability are for example highlighted by the compact rule set obtained for the prostate cancer dataset shown in Fig. 2. Apart from indicating the relevance of six used genes as putative biomarkers, the first two conjunctive rules also point to potential associations between their included genes. Corresponding genes which are frequently selected as informative features in rule sets across different cross-validation cycles and different ensemble base classifiers provide robust and informative predictors with regard to the outcome attribute. In this context, using a high number of base models combined to an ensemble can even be beneficial for data interpretation due to the variance-reducing effects of ensemble learning [62] which result in more robust statistics on the importance of single features in the predicates of the decision rules. This concept matches well with the results of both the automated text-mining analysis and the manual inspection of the literature, showing that in gene rankings obtained from BioHEL the top-ranked genes have all known or putative functional associations to the studied cancer diseases.

As a by-product of our experiments, we also compared the performance of different types of attribute selection methods: A univariate selection approach (PLSS [46]), a combinatorial filter (CFS [36]) and an embedded approach (RFS [38]). The combination of the predictors with the fast univariate PLSS approach provided unexpectedly high accuracies in comparison to the more complex CFS and RFS methods, however, PLSS lacks the adaptivity of the CFS approach, which is capable of automatically estimating the optimal number of selected features. Overall, the classification results obtained for different feature selection methods across all prediction methods and all the original datasets (and also the different pre-processing variants presented in the Material S1) suggest that the user should not rely on a single selection approach as a general method of choice. Instead, we recommend to use both the CFS approach, as the most adaptive multivariate approach, and the univariate PLSS approach, as the most successful approach for settings in which all or most of the selected features are univariately significant. Given the cross-validation results for both of these selection methods on the available labelled training data, the user can then choose the approach that provides the best performance in this validation. Moreover, applying both the CFS and PLSS method and comparing the results has the added benefit of enabling a distinction between features selected as univariately or multivariately significant.

Possible future extensions for the machine learning systems BioHEL and GAssist include integrating prior clinical or biological knowledge into the analysis and directly combining the system with automated literature mining tools to better exploit the information content of the generated models. On the whole, the performance reached in comparison to other benchmark predictors and the benefits in terms of interpretability and robust ranking of genes show that rule-based evolutionary machine learning algorithms can be profitably applied for the supervised analysis of microarray data.

## Supporting Information

Material S1 **Supplementary information on datasets and robustness statistics.** The Supplementary Material contains details on the source and normalization for each microarray dataset used in this study, cross-validation results for different dataset pre-processing methods and a robustness analysis for the feature selection methods.
(PDF)
