# Supplementary material for: Using Rule-Based Machine Learning for Candidate Disease Gene Prioritization and Sample Classification of Cancer Gene Expression Data
Source: PLoS One. 2012 Jul 11;7(7):e39932. doi: 10.1371/journal.pone.0039932 (PMC3394775; doi:10.1371/journal.pone.0039932)
Supplement: Material S1 — Supplementary information on datasets and robustness statistics. The Supplementary Material contains details on the source and normalization for each microarray dataset used in this study, cross-validation results for different dataset pre-processing methods and a robustness analysis for the feature selection methods. (PDF) [file pone.0039932.s001.pdf]

Using rule-based machine learning for candidate disease gene prioritization and sample classification of cancer gene expression data - Supplementary Material

Enrico Glaab, Jaume Bacardit, Jonathan M. Garibaldi, Natalio Krasnogor

Contents

1 Datasets and normalization **2**

2 Cross-validation results for different dataset pre-processing variants **3**

3 Robustness of feature selection methods **10**

1 Datasets and normalization

Diffuse large B-cell lymphoma (DLBCL)

The lymphoma dataset [1] contains expression values for 7,129 genetic probes and 77 microarray samples, 58 of which were obtained from patients suffering from diffuse large B-cell lymphoma (D), while the remaining samples are derived from a related B-cell lymphoma type, termed follicular lymphoma (F). The experiments in this microarray study have been carried out on an Affymetrix HU6800 oligonucleotide platform.

To pre-process the raw data, the *Variance stabilizing normalization* method [2] was applied to filter out intensity-dependent variance. This was done using the *vsn* library and the *expresso* package in the R statistical learning environment [3]. Moreover, a thresholding was applied based on the suggestions in the supplementary material of the original publication associated with the dataset [1], and a fold-change filter used to remove features with low variance (all genetic probe vectors with less than a 3-fold change between the maximum and minimum expression value were discarded), resulting in 2647 remaining genetic probes (the pre-processed data is available online: http://icos.cs.nott.ac.uk/datasets/microarray.html).

Prostate cancer

The prostate cancer dataset [4] consists of expression measurements for 12,600 genetic probes across 50 normal tissues and 52 prostate cancer tissues. All experiments have been carried out on Affymetrix Hum95Av2 arrays. Due to the large number of samples, the fast GeneChip RMA (GCRMA) normalization algorithm was applied [5], a method that combines stochastic and sequence-based physical models to estimate the mRNA abundances. Moreover, thresholding was employed based on the suggestions of the original publication associated with the dataset [4] and a fold change filter to remove all probes with less than a 2-fold change between the maximum and minimum expression value, providing 2135 remaining genetic probes (the pre-processed data is available online: http://icos.cs.nott.ac.uk/datasets/microarray.html). Moreover, to investigate the robustness of the prediction and feature selection results across different filtering settings (see sections 2 and 3 in the Suppl. Mat.), two additional versions of the prostate cancer data were obtained by applying a 3-fold change filter (providing 340 remaining genetic probes) and a 1.5-fold change filter (providing 7355 remaining genetic probes).

Breast cancer

The breast cancer dataset from the collaborating Queen's Medical Centre [6, 7, 8, 9] provides gene expression values for 128 primary breast tumors across 47,293 genetic probes. Two groups of tumor samples can be distinguished in the data, the luminal group (L, 84 samples), which is characterised by oestrogen receptor expression, and the non-luminal group (N, 44 samples, no oestrogen receptor expression). The expression profiling procedure has previously been described in detail [6, 7, 8], and has also been applied in a recent ensemble gene selection analysis of this dataset [9]. Since the probe level data was obtained from a Sentrix Human-6 BeadChip platform (v1.0, Illumina, San Diego, CA), the dedicated Bioconductor "beadarray" package was used for normalization and probe replicate summarization (the pre-processed data is available online: http://icos.cs.nott.ac.uk/datasets/microarray.html).

2 Cross-validation results for different dataset pre-processing variants

To analyze the robustness of BioHEL's sample classification performance across different settings for the pre-filtering of datasets and the maximum number of selected attributes, the whole experimental protocol as outlined in the main manuscript was applied again on six different pre-processed versions of the largest dataset (obtained from the prostate cancer study by Singh et al. [4]. For this purpose, first, two additional pre-filtered versions of the prostate cancer data were generated by removing all genetic probe vectors with less than a 3-fold change (resulting in 340 remaining genetic probes), and respectively a 1.5-fold change (resulting in 7355 remaining genetic probes), between the maximum and minimum expression value (see classification results for these datasets in Table 1 and 2). Moreover, the original pre-processed version of the prostate cancer dataset with a 2-fold filtering was used in combination with four different maximum numbers of selected features (5, 15, 50 and 100; see Tables 3, 4, 5 and 6). For all these input settings, the predictive performance was evaluated for each combination between the prediction methods (BioHEL, GAssist, RF, SVM, PAM) and the feature selection methods (CFS, RFS, PLSS) using both external 10-fold cross-validation (CV) and Leave-one-out CV (LOOCV), as described in the main manuscript.

Overall, BioHEL attained average cross-validated classification accuracies between 84% and 95% for all of the above settings, with robust results across all data pre-processing methods and similar performances in relation to the other benchmark approaches as compared to the default settings used in the main manuscript. The best average accuracies obtained with BioHEL for each combination of a dataset pre-processing with a cross-validation procedure (highlighted in bold face in the tables) were always above 90% and similar to the average accuracies obtained with the best alternative benchmark approach (in six cases, the same maximum performance was reached, in one case BioHEL was the only method reaching the highest average accuracy, and in the remaining cases BioHEL's highest average accuracy was within 3% of the best overall accuracy).

In summary, these results confirm that BioHEL's performance is robust and competitive in comparison to other widely used microarray classification approaches across different pre-filtering settings with and without feature selection.

Table 1: **10-fold cross-validation results for different pre-processed variants of the Prostate cancer dataset**

Dataset	Feature Selection	Classification	AVG (%)	STDDEV
3-fold pre-processing (30 features)	CFS	BioHEL	84	8
	CFS	GAssist	85	11
	CFS	SVM	90	9
	CFS	RF	89	12
	CFS	PAM	90	10
	PLSS	BioHEL	87	10
	PLSS	GAssist	90	11
	PLSS	SVM	85	19
	PLSS	RF	88	13
	PLSS	PAM	84	12
	**RFS**	**BioHEL**	**91**	**7**
	RFS	GAssist	89	9
	RFS	SVM	89	12
	**RFS**	**RF**	**94**	**8**
	RFS	PAM	88	13
	none	BioHEL	90	8
1.5-fold pre-processing (30 features)	CFS	BioHEL	90	8
	CFS	GAssist	88	11
	CFS	SVM	93	8
	CFS	RF	91	11
	CFS	PAM	93	9
	PLSS	BioHEL	90	9
	PLSS	GAssist	90	12
	PLSS	SVM	88	8
	PLSS	RF	94	8
	PLSS	PAM	93	10
	RFS	BioHEL	86	10
	RFS	GAssist	90	12
	RFS	SVM	90	8
	**RFS**	**RF**	**95**	**8**
	RFS	PAM	91	10
	**none**	**BioHEL**	**95**	**8**

10-fold cross-validation results obtained with BioHEL, SVM, RF and PAM and three feature selection methods (CFS, PLSS, RFS) on the prostate cancer dataset using two pre-processing variants (2-fold and 1.5-fold filtering); AVG = average accuracy, STDDEV = standard deviation; the highest accuracies achieved with BioHEL and the best alternative method are both shown in bold type for each dataset.

Table 2: **Leave-one-out cross-validation results for different pre-processing variants of the Prostate cancer dataset**

Dataset	Feature Selection	Classification	AVG (%)	STDDEV
3-fold pre-processing (30 features)	CFS	BioHEL	87	33
	CFS	GAssist	87	33
	CFS	SVM	90	30
	CFS	RF	88	32
	CFS	PAM	87	34
	PLSS	BioHEL	86	34
	PLSS	GAssist	90	30
	PLSS	SVM	82	32
	PLSS	RF	92	27
	PLSS	PAM	84	37
	**RFS**	**BioHEL**	**91**	**28**
	RFS	GAssist	92	27
	RFS	SVM	86	35
	**RFS**	**RF**	**93**	**25**
	RFS	PAM	83	37
	none	BioHEL	90	30
1.5-fold pre-processing (30 features)	CFS	BioHEL	91	28
	CFS	GAssist	86	34
	**CFS**	**SVM**	**95**	**22**
	CFS	RF	93	25
	CFS	PAM	94	24
	PLSS	BioHEL	90	30
	PLSS	GAssist	90	30
	PLSS	SVM	82	32
	PLSS	RF	94	24
	PLSS	PAM	93	25
	RFS	BioHEL	90	30
	RFS	GAssist	89	31
	RFS	SVM	90	30
	**RFS**	**RF**	**95**	**22**
	RFS	PAM	89	31
	**none**	**BioHEL**	**95**	**22**

Leave-one-out cross-validation results obtained with BioHEL, SVM, RF and PAM and three feature selection methods (CFS, PLSS, RFS) on the prostate cancer dataset using two pre-processing variants (2-fold and 1.5-fold filtering); AVG = average accuracy, STDDEV = standard deviation; the highest accuracies achieved with BioHEL and the best alternative method are both shown in bold type for each dataset.

Table 3: **10-fold cross-validation results for different maximum numbers of selected features on the Prostate cancer dataset (1)**

Dataset	Feature Selection	Classification	AVG (%)	STDDEV
	**CFS**	**BioHEL**	**93**	**6**
	CFS	GAssist	90	10
	**CFS**	**SVM**	**94**	**10**
	**CFS**	**RF**	**94**	**11**
	CFS	PAM	93	8
	PLSS	BioHEL	89	11
2-fold pre-processing - 5 features	PLSS	GAssist	89	12
	PLSS	SVM	92	8
	PLSS	RF	90	9
	PLSS	PAM	91	10
	RFS	BioHEL	88	6
	RFS	GAssist	85	12
	RFS	SVM	89	11
	RFS	RF	88	10
	RFS	PAM	87	9
	**CFS**	**BioHEL**	**93**	**8**
	CFS	GAssist	91	8
	CFS	SVM	93	9
	CFS	RF	92	11
	**CFS**	**PAM**	**94**	**8**
	PLSS	BioHEL	91	7
2-fold pre-processing - 15 features	PLSS	GAssist	93	9
	PLSS	SVM	88	10
	PLSS	RF	90	10
	PLSS	PAM	90	11
	RFS	BioHEL	91	8
	RFS	GAssist	90	11
	RFS	SVM	90	11
	RFS	RF	93	9
	RFS	PAM	91	10

10-fold cross-validation results obtained with BioHEL, SVM, RF and PAM and three feature selection methods (CFS, PLSS, RFS) on the prostate cancer dataset using alternative settings for the maximum number of selected features (5 and 15, see next page for 50 and 100 maximum features); AVG = average accuracy, STDDEV = standard deviation; the highest accuracies achieved with BioHEL and the best alternative method are both shown in bold type for each dataset.

Table 4: **10-fold cross-validation results for different maximum numbers of selected features on the Prostate cancer dataset (2)**

Dataset	Feature Selection	Classification	AVG (%)	STDDEV
	**CFS**	**BioHEL**	**93**	**8**
	CFS	GAssist	92	8
	CFS	SVM	93	9
	CFS	RF	92	11
	**CFS**	**PAM**	**94**	**8**
	**PLSS**	**BioHEL**	**93**	**8**
2-fold	PLSS	GAssist	93	8
pre-processing	PLSS	SVM	89	9
- 50 features	PLSS	RF	93	9
	PLSS	PAM	89	11
	RFS	BioHEL	91	8
	RFS	GAssist	92	11
	RFS	SVM	91	11
	RFS	RF	93	9
	RFS	PAM	91	10
	CFS	BioHEL	93	8
	CFS	GAssist	91	8
	CFS	SVM	93	9
	CFS	RF	92	11
	**CFS**	**PAM**	**94**	**8**
	**PLSS**	**BioHEL**	**94**	**7**
2-fold	PLSS	GAssist	91	12
pre-processing	PLSS	SVM	90	9
100 features	PLSS	RF	92	9
	PLSS	PAM	89	11
	RFS	BioHEL	91	7
	RFS	GAssist	91	12
	RFS	SVM	87	13
	RFS	RF	93	9
	RFS	PAM	91	10

10-fold cross-validation results obtained with BioHEL, SVM, RF and PAM and three feature selection methods (CFS, PLSS, RFS) on the prostate cancer dataset using alternative settings for the maximum number of selected features (50 and 100, see previous page for 5 and 15 maximum features); AVG = average accuracy, STDDEV = standard deviation; the highest accuracies achieved with BioHEL and the best alternative method are both shown in bold type for each dataset.

Table 5: **Leave-one-out cross-validation results for different maximum numbers of selected features on the Prostate cancer dataset (1)**

Dataset	Feature Selection	Classification	AVG (%)	STDDEV
	CFS	BioHEL	90	30
	CFS	GAssist	88	32
	**CFS**	**SVM**	**94**	**24**
	CFS	RF	92	27
	CFS	PAM	92	27
	PLSS	BioHEL	89	31
2-fold	PLSS	GAssist	90	30
pre-processing	PLSS	SVM	92	27
- 5 features	PLSS	RF	90	30
	PLSS	PAM	90	30
	**RFS**	**BioHEL**	**94**	**24**
	RFS	GAssist	89	31
	RFS	SVM	92	27
	RFS	RF	88	32
	RFS	PAM	92	27
	CFS	BioHEL	91	28
	CFS	GAssist	91	28
	CFS	SVM	93	25
	**CFS**	**RF**	**93**	**25**
	CFS	PAM	92	27
	**PLSS**	**BioHEL**	**94**	**24**
2-fold	PLSS	GAssist	91	28
pre-processing	PLSS	SVM	91	29
- 15 features	PLSS	RF	91	29
	PLSS	PAM	91	29
	RFS	BioHEL	89	31
	RFS	GAssist	89	31
	RFS	SVM	90	30
	**RFS**	**RF**	**93**	**25**
	RFS	PAM	92	27

Leave-one-out cross-validation results obtained with BioHEL, SVM, RF and PAM and three feature selection methods (CFS, PLSS, RFS) on the prostate cancer dataset using alternative settings for the maximum number of selected features (5 and 15, see next page for 50 and 100 maximum features); AVG = average accuracy, STDDEV = standard deviation; the highest accuracies achieved with BioHEL and the best alternative method are both shown in bold type for each dataset.

Table 6: **Leave-one-out cross-validation results for different maximum numbers of selected features on the Prostate cancer dataset (2)**

Dataset	Feature Selection	Classification	AVG (%)	STDDEV
	CFS	BioHEL	89	31
	CFS	GAssist	89	31
	**CFS**	**SVM**	**93**	**25**
	**CFS**	**RF**	**93**	**25**
	CFS	PAM	92	27
	**PLSS**	**BioHEL**	**93**	**25**
2-fold	**PLSS**	**GAssist**	**93**	**25**
pre-processing	PLSS	SVM	91	29
- 50 features	PLSS	RF	92	27
	PLSS	PAM	91	29
	RFS	BioHEL	89	31
	**RFS**	**GAssist**	**93**	**25**
	**RFS**	**SVM**	**93**	**25**
	RFS	RF	92	27
	RFS	PAM	90	30
	CFS	BioHEL	91	28
	CFS	GAssist	89	31
	**CFS**	**SVM**	**93**	**25**
	**CFS**	**RF**	**93**	**25**
	CFS	PAM	92	27
	**PLSS**	**BioHEL**	**93**	**25**
2-fold	**PLSS**	**GAssist**	**93**	**25**
pre-processing	PLSS	SVM	90	30
- 100 features	PLSS	RF	92	27
	PLSS	PAM	91	29
	**RFS**	**BioHEL**	**93**	**25**
	RFS	GAssist	92	27
	RFS	SVM	91	29
	**RFS**	**RF**	**93**	**25**
	RFS	PAM	91	29

Leave-one-out cross-validation results obtained with BioHEL, SVM, RF and PAM and three feature selection methods (CFS, PLSS, RFS) on the prostate cancer dataset using alternative settings for the maximum number of selected features (50 and 100, see previous page for 5 and 15 maximum features); AVG = average accuracy, STDDEV = standard deviation; the highest accuracies achieved with BioHEL and the best alternative method are both shown in bold type for each dataset.

3 Robustness of feature selection methods

In addition to the comparison of the classification results across different feature selection, prediction, cross-validation (CV) and pre-processing methods, we also investigated the stability of the feature selection results across different CV-cycles, using BioHEL both with and without external attribute selection on the smallest and largest pre-filtered version of the prostate cancer data by Singh et al. [4]). For this purpose, for each 10-fold CV-cycle the 10 top-ranked selected genetic probes were determined, and the number of times they were chosen across at least 8 of 10 CV-cycles was recorded (these numbers are reported as robustness scores in Tables 7 and 8). The highest scores (5 and 6) were obtained when using BioHEL without external feature selection method. However, in combination with some of the external selection methods (e.g. PLSS) the same or similar robustness scores were reached. RFS tended to provide slightly lower scores than the other approaches, including a score of 0 in one case (for the 1.5-fold filtering of the prostate cancer dataset, providing the largest number of 7355 remaining genetic probes), which might result from the stochastic nature of the random forests approach.

When comparing the attribute identifiers for the 10 top-ranked features obtained from BioHEL using no external selection with those obtained directly from PLSS, RFS and CFS (prior to the classification), several features that were shared across different CV-cycles for a single selection method were also shared across these different algorithms (see Tables 9, 10 and 11 for the results on the 1.5-fold, 2-fold and 3-fold pre-filtered version of the prostate cancer dataset; attributes which appear at least twice across these tables are highlighted by matching colors). On all pre-processed versions of the prostate cancer dataset BioHEL shares at least its first 4 top-ranked features with at least one of the external feature selection methods. Given that only the 10 highest-ranked features are considered for each method in this comparison and the initial dataset contained between 7355 and 340 attributes (for 1.5-fold and 3-fold filtering, respectively), these results reveal a significant concordance between the top-ranked features for different methods. Moreover, since most of these high-ranked attributes across different methods were also selected by the univariate PLSS approach, the corresponding features are univariately significant, with four notable exceptions: The genetic probes 37068_at (*phospholipase A2, group VII*), 38291_at (*proenkephalin*), 914_g_at (*transcriptional regulator ERG*) and 38634_at (*cellular retinol binding protein 1*) appear among the top 10 attributes for multiple multivariate selection methods but never among the top-ranked features for PLSS. This might indicate that these attributes are selected by the multivariate approaches due to their significance in the presence of other features (since the outcome variable is binary, a meaningful comparison of the correlation between these features and the outcome was not possible).

A more specific analysis of the individual shared and non-shared genetic probes across the different selection methods was not within the scope of this study; however, in the main manuscript a detailed discussion of the top-ranked attributes selected by the more robust ensemble feature selection method (Ensemble FS) and BioHEL-based feature ranking (BioHEL FR) is provided in the literature mining section.

Table 7: **Feature selection robustness scores on the prostate cancer dataset (1)**

	3-fold pre-processing	1.5-fold pre-processing
CFS	4	5
PLSS	5	6
RFS	5	0
noFS	6	5

Feature selection robustness scores across 10 cross-validation cycles on the prostate cancer dataset, corresponding to the number of features among the 10 top-ranked attributes that were selected across at least 8 out of 10 cycles. The results are shown both for the smallest and largest version of the prostate cancer dataset (3-fold and 1.5-fold pre-filtering) using the same settings as for the corresponding classification results reported in Table 1 (for the other pre-processing variants of this dataset, see Table 8).

Table 8: **Feature selection robustness scores on the prostate cancer dataset (2)**

	5 features	15 features	50 features	100 features
CFS	5	5	5	5
PLSS	4	4	6	4
RFS	3	3	3	2

Feature selection robustness scores across 10 cross-validation cycles on the prostate cancer dataset, corresponding to the number of features among the 10 top-ranked attributes that were selected across at least 8 out of 10 cycles. The results are shown for the 2-fold pre-processing of the prostate cancer dataset using different maximum numbers of selected features in the external attribute selection (5, 15, 50 and 100 - see also Tables 3 and 4 for the corresponding classification results).

Table 9: **Comparison of the 10 top-ranked features for different feature selection methods and BioHEL without external selection on the prostate cancer dataset (1.5-fold pre-processing)**

BioHEL	PLSS	RFS	CFS
32598_at	37639_at	37639_at	37639_at
37068_at	41468_at	41706_at	32598_at
37639_at	41706_at	32598_at	2041_i_at
41706_at	1740_g_at	37068_at	31791_at
914_g_at	38827_at	36174_at	38087_s_at
103_at	37366_at	34730_g_at	33121_g_at
38028_at	40282_s_at	39315_at	36928_at
38803_at	32598_at	40024_at	37366_at
38951_at	38087_s_at	35776_at	33883_at
39939_at	38634_at	41732_at	38326_at

Comparison of the 10 top-ranked genetic probes for the external selection methods (without machine learning) and for BioHEL (without external attribute selection) for a 1.5-fold pre-filtering of the prostate cancer dataset and 10-fold CV. Features occurring at least twice across this table and table 10 and 11 are colored, and shared features receive the same colors across all tables.

Table 10: **Comparison of the 10 top-ranked features for different feature selection methods and BioHEL without external selection on the prostate cancer dataset (2-fold pre-processing)**

BioHEL	PLSS	RFS	CFS
32598_at	37639_at	41706_at	37639_at
37639_at	41468_at	37639_at	32598_at
40282_s_at	41706_at	38028_at	38087_s_at
41706_at	40282_s_at	32598_at	31791_at
32250_at	1661_i_at	38291_at	39756_g_at
38028_at	1740_g_at	37720_at	31906_at
914_g_at	34775_at	914_g_at	33825_at
37068_at	1662_r_at	38057_at	37599_at
41741_at	37366_at	575_s_at	914_g_at
36627_at	32598_at	41468_at	35178_at

Comparison of the 10 top-ranked genetic probes for the external selection methods (without machine learning) and for BioHEL (without external attribute selection) for a 2-fold pre-filtering of the prostate cancer dataset and 10-fold CV. Features occurring at least twice across this table and tables 9 and 11 are colored, and shared features receive the same colors across all tables.

Table 11: **Comparison of the 10 top-ranked features for different feature selection methods and BioHEL without external selection on the prostate cancer dataset (3-fold pre-processing)**

BioHEL	PLSS	RFS	CFS
37068_at	41706_at	41706_at	41706_at
38291_at	1740_g_at	38291_at	40282_s_at
38634_at	38827_at	38634_at	38827_at
40282_s_at	40282_s_at	37068_at	38634_at
41483_s_at	34775_at	40282_s_at	37068_at
41706_at	1661_i_at	38827_at	38291_at
36627_at	291_s_at	40071_at	36491_at
38127_at	38469_at	34050_at	1740_g_at
AFFX-M27830_5_at	36491_at	33415_at	40071_at
1612_s_at	38604_at	34775_at	39154_at

Comparison of the 10 top-ranked genetic probes for the external selection methods (without machine learning) and for BioHEL (without external attribute selection) for a 3-fold pre-filtering of the prostate cancer dataset and 10-fold CV. Features occurring at least twice across this table and table 9 and 10 are colored, and shared features receive the same colors across all tables.

Supplementary Materials - References

[1] Shipp M, Ross K, Tamayo P, Weng A, Kutok J, Aguiar R, Gaasenbeek M, Angelo M, Reich M, Pinkus G, Ray T, Koval M, Last K, Norton A, Lister T, Mesirov J, Neuberg D, Lander E, Aster J, Golub T: **Diffuse large B-cell lymphoma outcome prediction by gene-expression profiling and supervised machine learning**. *Nat. med.* 2002, **8**:68–74.

[2] Huber W, von Heydebreck A, Sültmann H, Poustka A, Vingron M: **Variance stabilization applied to microarray data calibration and to the quantification of differential expression**. *Bioinformatics* 2002, **18**:96–104.

[3] Ihaka R, Gentleman R: **R: A Language for Data Analysis and Graphics**. *J Comput Graph Stat* 1996, **5**(3):299–314.

[4] Singh D, Febbo P, Ross K, Jackson D, Manola J, Ladd C, Tamayo P, Renshaw A, D'Amico A, Richie J, Lander E, Loda M, Kantoff P, Golub T, Sellers W: **Gene expression correlates of clinical prostate cancer behavior**. *Cancer Cell* 2002, **1**(2):203–209.

[5] Wu Z, Irizarry R: **Stochastic Models Inspired by Hybridization Theory for Short Oligonucleotide Arrays**. *J Comput Biol* 2005, **12**(6):882–893.

[6] Chin S, Teschendorff A, Marioni J, Wang Y, Barbosa-Morais N, Thorne N, Costa J, Pinder S, van de Wiel M, Green A, Ellis I, Porter P, Tavaré S, Brenton J, Ylstra B, Caldas C: **High-resolution aCGH and expression profiling identifies a novel genomic subtype of ER negative breast cancer**. *Genome Biol.* 2007, **8**(10):R215.

[7] Naderi A, Teschendorff A, Barbosa-Morais N, Pinder S, Green A, Powe D, Robertson J, Aparicio S, Ellis I, Brenton J, Caldas C: **A gene-expression signature to predict survival in breast cancer across independent data sets**. *Oncogene* 2006, **26**(10):1507–1516.

[8] Zhang H, Rakha E, Ball G, Spiteri I, Aleskandarany M, Paish E, Powe D, Macmillan R, Caldas C, Ellis I, Green A: **The proteins FABP7 and OATP2 are associated with the basal phenotype and patient outcome in human breast cancer**. *Breast Cancer Res. Treat.* 2010, **121**:41–51.

[9] Habashy HO, Powe DG, Glaab E, Krasnogor N, Garibaldi JM, Rakha EA, Ball G, Green AR, Caldas C, Ellis IO: **RERG (Ras-related and oestrogen-regulated growth-inhibitor) expression in breast cancer: A marker of ER-positive luminal-like subtype**. *Breast Cancer Research and Treatment* 2011, **128**(2):315–326.
